# Synaptic Injury in the Thalamus Accompanies White Matter Injury in Hypoxia/Ischemia-Mediated Brain Injury in Neonatal Rats

**DOI:** 10.1155/2019/5249675

**Published:** 2019-10-09

**Authors:** Na Liu, Xin Tong, Wanjie Huang, Jianhua Fu, Xindong Xue

**Affiliations:** Department of Pediatrics, Shengjing Hospital of China Medical University, Shenyang 110004, China

## Abstract

The broad spectrum of disabilities caused by white matter injury (WMI) cannot be explained simply by hypomyelination. Synaptic injury in the thalamus may be related to disabilities in WMI survivors. Neuronal injury in the thalamus has been found most commonly in autopsy cases of preterm WMI. We hypothesized that hypoxia/ischemia (HI) in neonatal rats results in synaptic abnormalities in the thalamus that contribute to disabilities in WMI survivors. We examined changes in synapses in a neonatal rat model of HI-induced WMI. Right common carotid artery ligation and hypoxia (8% oxygen for 2.5 hours (h)) were performed in three-day-old Sprague-Dawley rats. We found HI rats performed worse in the Morris water maze test than sham rats, suggesting long-term cognition impairment after HI injury. A loss of synapses in the thalamus accompanied by hypomyelination and oligodendrocytes (OLs) reduction was observed. At the ultrastructural level, reductions in active zone (AZ) length and postsynaptic density (PSD) thickness were detected at 2 weeks after HI exposure. Furthermore, increased expression of synaptophysin and PSD-95 in both groups was observed from 3 days (d) to 21 d after hypoxic/ischemic (HI) injury. PSD-95 expression was significantly lower in HI rats than in sham rats from 14 d to 21 d after HI injury, and synaptophysin expression was significantly lower in HI rats from 7 d to 14 d after HI injury. However, no significant difference in synaptophysin expression was observed between HI rats and sham rats at 21 d after HI injury. The results demonstrated synaptic abnormalities in the thalamus accompanied by hypomyelination in WMI in response to HI exposure, which may contribute to the diverse neurological defects observed in WMI patients. Although synaptic reorganization occurred as a compensatory response to HI injury, the impairments in synaptic transmission were not reversed.

## 1. Introduction

Cerebral white matter injury (WMI) is the leading contributor to neurodevelopmental disabilities in premature infants. Because of improvements in birth and survival rates for premature births in recent years, neurodevelopmental disabilities in survivors have become more prominent [1–3]. In the USA, the neurodevelopmental disabilities for premature neonates with a birth weight less than 1500 g include cerebral palsy in approximately 10%–15% of the survivors and behavioral deficits in approximately 25–50% of the survivors, including cognitive decline [2, 4]. Because WMI leads to adverse outcomes and a great social burden, efforts have been made to understand the disease and to reverse brain injury.

Hypomyelination resulted from disrupted synthesis of myelin after injury is commonly found in WMI survivors, especially in diffuse WMI survivors [5–8]. Oligodendrocytes (OLs) degeneration and dysmaturation may contribute to the pathogenesis of hypomyelination [1, 5, 6, 8–10]. Premyelinating oligodendrocytes (pre-OLs), which have been reported to be highly susceptible to HI and inflammation, have been shown to be the main cell target in WMI [3, 5, 9, 10]. The broad spectrum of disabilities caused by WMI, including behavioral deficits (e.g., cognitive, attentional, and motor deficits), appears to be related to not simply myelination failure. The neuroanatomic substrate of diverse disabilities of WMI survivors remains unclear. The thalamus is related to cognition via extensive connections with the cerebral cortex [11]. Evidence of neuron loss has been found in autopsy cases of periventricular leukomalacia (PVL), a severe form of preterm WMI [12]. Neurons in the thalamus are most commonly affected in WMI infants [12], potentially contributing to the diverse spectrum of neurological impairments [7, 13]. Neuroimaging studies indicated a diminished volume of the thalamus in long-term survivors with preterm WMI [14]. Thus, we chose the thalamus as a typical area for research.

Synapses are formed between different neurons and act as the basic unit for information transfer in the brain. Synaptic connections are essential for the organization of the complicated network in the human brain [15, 16]. Due to their important foundation for interneuronal connection, synapse loss and synaptic dysfunction lead to a series of disorders, including cognitive, learning, attentional, and motor deficits [16, 17]. Synaptogenesis and plasticity are important in immature brain development [16]. Dysfunction and loss of synapses during immature brain development can result in diverse neurological sequelae, including cognitive, learning, attentional, and motor deficits. Synapse degeneration can be found in the animal model of hypoxic/ischemic (HI) encephalopathy [18], and alterations in the function and expression of glutamate receptors during the neonatal period, which are components of postsynaptic elements, may contribute to future epileptogenesis [19].

Synapses are formed between neurons. Due to evidence pointing towards neuron injuries in the thalamus of WMI patients and the potential relationship between synaptic dysfunction and the neurological sequelae of WMI survivors, we hypothesized that WMI patients may have synaptic injuries in the thalamus. In this study, we used a neonatal rat model of WMI induced by HI injury to investigate synaptic injuries in the thalamus.

## 2. Materials and Methods

### 2.1. Animals

All animal experiments were approved by the Animal Ethical Committee of China Medical University, Shenyang, China (2017PS140K). The animal model for WMI was induced according to the method provided by Vannucci et al. [20]. The surgery was performed following inhalation anesthesia. The right common carotid artery of 3-day-old (P3) Sprague-Dawley rats was double-ligated after inhalation of anesthesia, and a cut was made between the ligatures as described previously [21]. After the pups were allowed to recover for 1 hour (h), they were exposed to 2.5 h of hypoxia (8% O_2_/92% N_2_) in a container submerged in a water bath (37°C) to produce HI injury. After hypoxia exposure, all pups were sent back to their dams until they were sacrificed. The rats in the sham group were only exposed to right common carotid artery isolation without ligation or hypoxia exposure.

### 2.2. Behavioral Study

The Morris water maze was performed as a behavioral test on days P28–P33 (*n* = 10 each group). The facilities consisted of a circular black tank (160 cm in diameter and 60 cm in depth) and a platform (12 cm in diameter) in the first quadrant. The tank was filled with water (24 ± 0.5°C) to a level of 1.5 cm above the top surface of the platform. The navigation test was conducted four times daily for five consecutive days. Rats were placed randomly into the water at each of the four quadrants, facing the wall of the pool. A rat that succeeded in finding the platform within 60 s was allowed to stay on the platform for 20 s. The time taken to find the platform was defined as escape latency. A rat that failed to find the platform within 60 s was guided to the platform and allowed to stay there for 20 s. On day 6, the platform was removed and the probe test was conducted. The rat was placed at the opposite quadrant and allowed to swim for 60 s. The data were recorded by a video tracking system (Shanghai Mobile Datum Ltd, Shanghai City, China). All of the tests were performed by researchers who were blinded to the experimental group.

### 2.3. Immunohistochemistry

Rats were euthanized at 7 or 14 days (d) after HI injury and perfused with 4% paraformaldehyde in 0.1 M phosphate buffer before brains were harvested (*n* = 5 each group). The brains were then fixed in a 4% paraformaldehyde solution for 24 h at 4°C and embedded in paraffin. Coronal sections (3 *µ*m) were cut (HM340E; Thermo Fisher Microm, Walldorf, Germany). Tissue sections from brains harvested at 14 d after HI injury were sequentially deparaffinized in xylene and rehydrated in gradient ethanol solutions. Then, the sections were pretreated with heat-mediated antigen retrieval (EDTA-based pH 9.0 solution) for 20 min. Sections were treated with 3% hydrogen peroxide for 20 min at 37°C and blocked with goat serum for 30 min at 37°C. Sections were incubated at 4°C overnight with primary antibody (mouse anti-postsynaptic density (PSD)-95 monoclonal antibody, 1 : 200, Abcam, USA), followed by incubation with secondary antibody and streptavidin-horseradish peroxidase for 20 min at 37°C. Finally, the sections were developed with 3,3′-diaminobenzidine and then dehydrated and mounted in neutral balsam. Images were visualized and photographed by a light microscope (Olympus Corporation, Tokyo, Japan).

### 2.4. Immunofluorescence

After deparaffinization and heat-mediated antigen retrieval (EDTA-based pH 9.0 solution), as previously described, the tissue sections were blocked with goat serum for 30 min at 37°C and then incubated at 4°C overnight with primary antibody (mouse anti-myelin basic protein (MBP) monoclonal antibody, 1 : 400, Abcam, USA; rabbit anti-synaptophysin monoclonal antibody, 1 : 100, Abcam, USA; goat anti-Olig2 polyclonal antibody, 1 : 50, R&D, USA). Sections were washed three times with 0.1 M phosphate buffered saline (PBS) for 5 min before incubation with secondary antibodies (donkey anti-rabbit IgG, 1 : 500, Abcam, USA; donkey anti-mouse IgG, 1 : 500, Abcam, USA; donkey anti-goat IgG, 1 : 200, Abcam, USA) for 4 h at room temperature and 4′,6-diamidino-2-phenylindole (DAPI; 1 : 500, Sigma-Aldrich) sequentially for nuclear staining. Immunofluorescent images were visualized and photographed using a confocal laser-scanning microscope (C1; Nikon, Tokyo, Japan).

### 2.5. Western Blotting

At 3, 7, 14, and 21 d after HI injury, rats were euthanized, and brains were harvested (*n* = 5 each group). Thalamic tissues were isolated from the right hemispheres on ice and stored at −80°C. Total protein was extracted from frozen thalamic tissue samples and denatured. Protein samples (50 *µ*g) were separated by 4%–20% sodium dodecyl sulfate-polyacrylamide precast gel electrophoresis (Genscript, USA; 140 V for 1 h) and then transferred to polyacrylamide difluoride (PVDF) membranes (100 V for 60 min). Membranes were blocked in 5% nonfat milk for 2 h before overnight incubation at 4°C with primary antibody (mouse anti-PSD-95 monoclonal antibody, 1 : 1000, Abcam, USA; rabbit anti-synaptophysin monoclonal antibody, 1 : 5000, Abcam, USA; mouse anti-tubulin monoclonal antibody, 1 : 2000, Proteintech, USA). Then, the membranes were incubated with the appropriate secondary antibody (horseradish peroxidase-conjugated goat anti-rabbit or anti-mouse antibody, 1 : 5000; Proteintech, USA) and developed by enhanced chemiluminescence reagents (Thermo Scientific Pierce; Thermo Fisher Scientific, Waltham, USA). The intensities for all the bands were analyzed by ImageJ software and were normalized to the tubulin signal.

### 2.6. Electron Microscopy

At 14 d after HI injury, rats (*n* = 5 each group) were euthanized, brains were dissected, and thalamic tissue sections from the right hemispheres were cut to a size of approximately 1 mm^3^ at 4.0 to 5.0 mm posterior to bregma. Tissue samples were postfixed with 4% paraformaldehyde and 2.5% glutaraldehyde. Then, brain samples were treated in 1% osmium tetroxide, dehydrated in an acetone series, and embedded with epoxy resin. The tissue blocks were cut into ultrathin sections with an ultramicrotome and stained with uranyl acetate and lead citrate. Images were observed using a transmission electron microscope (JEM-1400; JEOL, Tokyo, Japan). Images were randomly selected and analyzed using ImageJ software.

### 2.7. Statistics

All the data from each experiment are represented as the mean ± SEM. GraphPad Prism 5 was used to create graphs and perform statistical analyses. Any significant differences between two groups were assessed by the *t*-test. The escape latency was analyzed using two-way analysis of variance for repeated measurements. The differences were considered statistically significant at *P* < 0.05.

## 3. Results

### 3.1. Impaired Cognition after Exposure to HI

**Figure 1:**
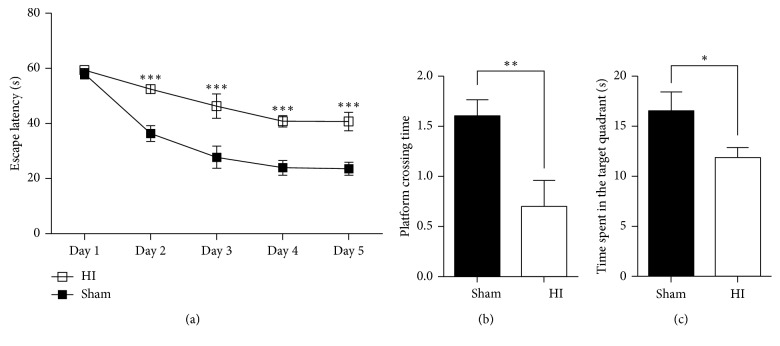
Cognitive function assessment by the Morris water maze test. (a) During the navigation test, HI rats showed a significantly longer escape latency compared to the sham rats from the 2nd day to the 5th day. In the probe test, the HI rats showed less platform crossing (b) and spent less time in the target quadrant (c) compared to the sham rats (^*∗*^*P* < 0.05, ^*∗∗*^*P* < 0.01, and ^*∗∗∗*^*P* < 0.001). Two-way analysis of variance (ANOVA) for repeated measurements was used to determine the difference of escape latency. Student's *t*-test was performed to determine the differences in the number of platform crossing and time spent in the target quadrant.

The Morris water maze is widely used for the assessment of spatial learning and memory, which are related to cognitive function [22]. In the navigation test, during the first 5 days, significantly longer escape latency was observed in rats from the HI group than the sham group (Figure 1(a), *P* < 0.001, at 2nd, 3rd, 4^th^, and 5th days). In addition, in the probe trial, rats in the HI group passed less frequently through the platform location (*P* < 0.01) and spent less time in the target quadrant (*P* < 0.05) compared to the sham group (Figures 1(b) and 1(c)), indicating the long-term cognitive deficits of HI rats.

### 3.2. OL Reduction and Hypomyelination Induced by HI Injury

**Figure 2:**
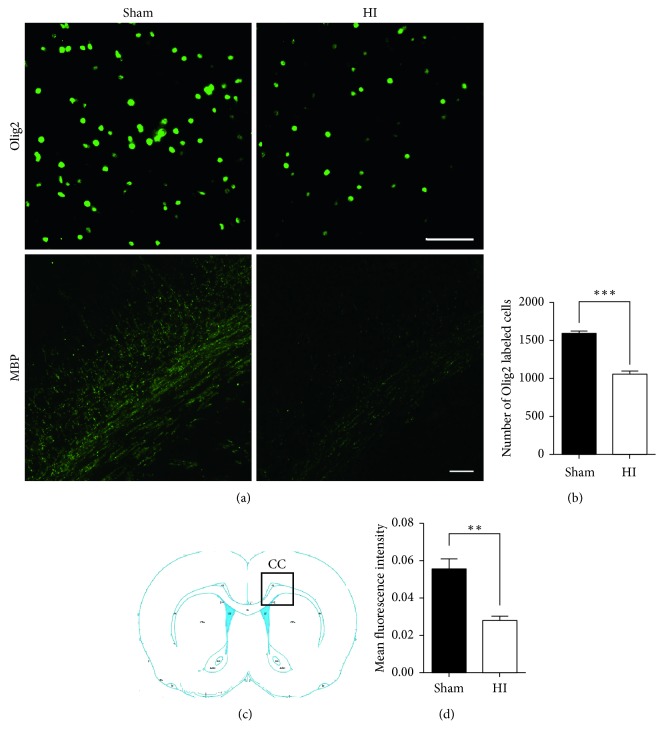
The changes in number of oligodendrocytes and myelination. (a, b) Olig2+ cells in immunofluorescence staining referred to oligodendrocytes (scale bar = 50 *μ*m). The quantitative analysis showed decreased number of oligodendrocytes (per mm^2^) in subcortical white matter of right hemispheres in HI rats at 7 d after HI injury compared with sham rats. (c) Diagram of the brain region analyzed for the expression of MBP. (a, d) Immunofluorescence staining showed MBP in corpus callosum (CC) axons in the right hemispheres (boxed area in (c)) at 14 d after HI injury (scale bar = 50 *μ*m). HI rats exhibited less myelination of CC axons in the right hemispheres than sham rats did. The projection fibers oriented to the cortex in the boxed area were hypomyelinated in HI rats (^*∗∗*^*P* < 0.01 and ^*∗∗∗*^*P* < 0.001).

We performed immunofluorescent staining of subcortical white matter with Olig2 at 7 d after HI injury to determine the distribution of Olig2-labeled OLs. As shown in Figures 2(a) and 2(b), the density of Olig2-labeled OLs in the subcortical white matter of right hemispheres was lower in HI rats than in sham rats, which was related to the myelination disturbance in white matter after HI injury.

To determine the effect of HI injury on myelination, we performed immunofluorescent staining for MBP, a myelin protein. Corpus callosum (CC) is rich in myelin in the periventricular region. It is widely used as a proxy of hypomyelination in perinatal WMI. At 14 d after HI injury, HI rats exhibited less myelination of CC axons in the right hemispheres than did sham rats (Figures 2(a), 2(c), and 2(d)). The projection fibers oriented to the cortex in the boxed area were hypomyelinated in HI rats, indicating reduced velocity of impulse conduction in this area in HI rats compared to that in sham rats.

### 3.3. Synaptic Changes Induced by HI Injury

We explored the synaptic changes caused by HI injury at the ultrastructural and biochemical levels.

#### 3.3.1. Changes in the Structure and Numerical Density of Synapses in the Thalamus after HI Injury

Sections of the thalamus at 14 d after HI injury were analyzed by electron micrography. Changes in the morphology and density of synapses were investigated at the ultrastructural level.

**Figure 3:**
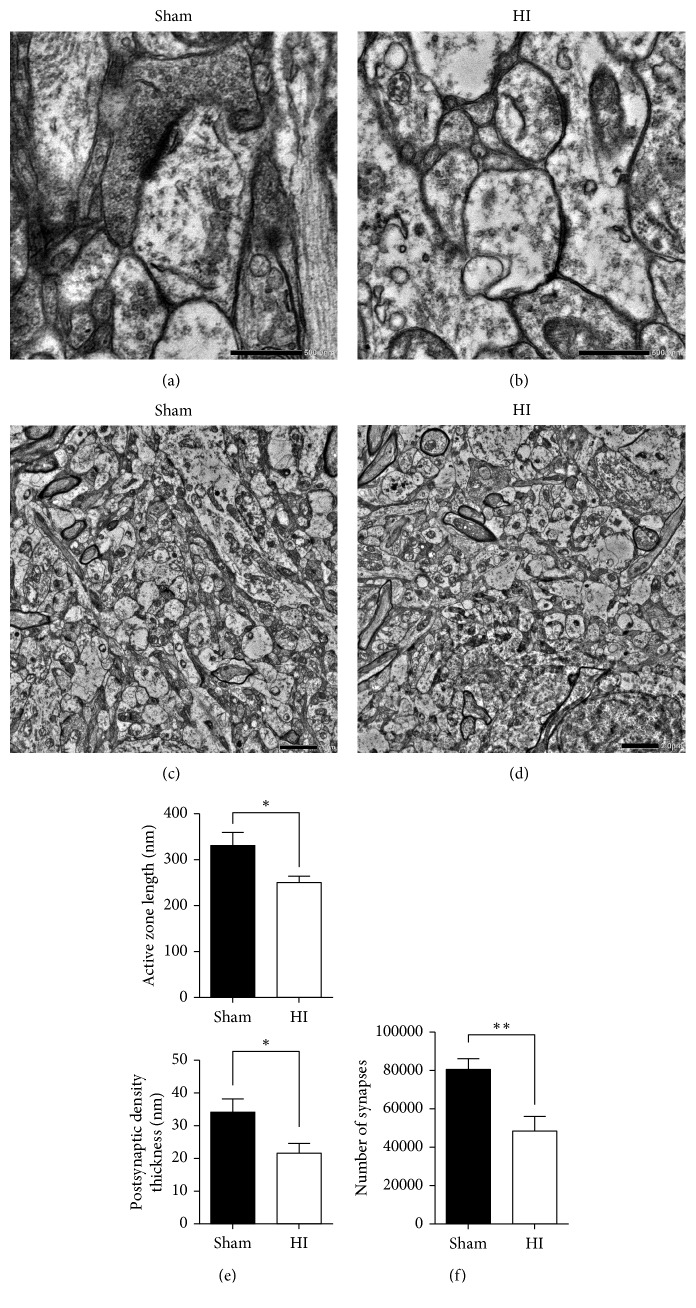
Ultrastructural changes observed in synapses at 14 d after HI injury in the thalamus. (a, b) TEM images of the synapses in the thalamus from the right hemispheres exhibited fewer synaptic vesicles at the presynaptic terminal in the HI group, accompanied by a decrease in vesicles located in the AZ and ready to release (scale bar = 500 nm). (c, d) Low magnification of TEM images of the synapses in the thalamus from the right hemispheres recognizable by their postsynaptic density (scale bar = 2 *µ*m). (e) The quantitative analysis showed that the AZ length and the PSD thickness were both decreased in the HI group compared with the sham group at 14 d after HI injury in the right thalamus. (f) The quantitative analysis of the numerical synaptic density of synapses per mm^2^ showed significant decrease of synapses in the right thalamus in the HI group at 14 d after HI injury (^*∗*^*P* < 0.05 and ^*∗∗*^*P* < 0.01).

The active zone (AZ), which is located in the presynaptic membrane, is considered to be involved in transmitter release. The PSD is a series of proteins in the postsynaptic membrane that plays a crucial role in signal exchange between neurons. We analyzed changes in AZ length and PSD thickness of synapses induced by HI injury. As shown in Figures 3(a)–3(e), compared with the sham group, the HI group exhibited a decrease in both AZ length and PSD thickness.

Compared to the sham group, the HI group exhibited fewer synaptic vesicles at the presynaptic terminal accompanied by a decrease in vesicles located in the AZ and ready to release (Figures 3(a) and 3(b)).

In the thalamus, the numerical density of synapses in the HI group (48,343 ± 7,621) was obviously lower than that in the sham group (80,486 ± 5,675, Figure 3(f)).

#### 3.3.2. Changes in Presynaptic and Postsynaptic Protein Expression

**Figure 4:**
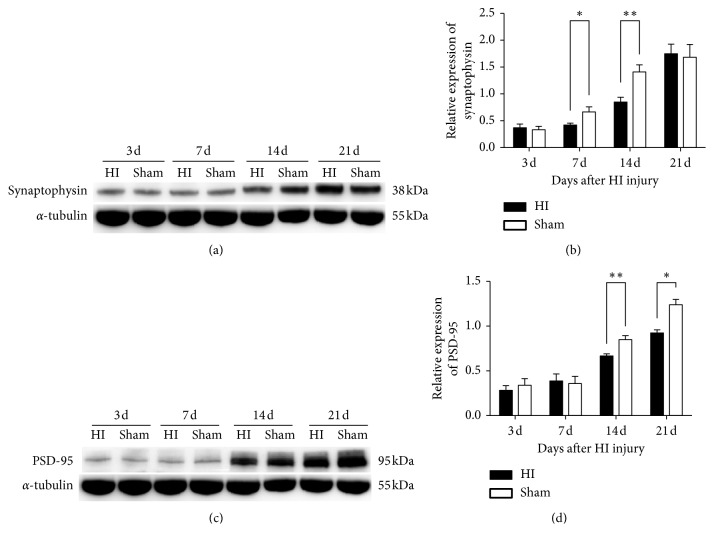
Changes in pre- and postsynaptic protein expression induced by WMI in the thalamus. (a, b) Presynaptic marker synaptophysin expression in the thalamus from the right hemispheres was significantly lower than the sham group at 7 d and 14 d after HI injury. (c, d) The level of postsynaptic marker PSD-95 was decreased at 14 and 21 d after HI injury in the HI group. *α*-Tubulin served as a loading control (^*∗*^*P* < 0.05 and ^*∗∗*^*P* < 0.01).

To examine the differential expression of presynaptic and postsynaptic proteins in the thalamus induced by HI injury, we performed western blotting analysis. After HI exposure, both synaptophysin and PSD-95 showed increasing trends in expression from 3 d to 21 d after HI injury, which indicated continuous synaptogenesis (Figures 4(a)–4(d)). The level of synaptophysin was not significantly different at 3 d after HI injury between the HI and sham groups. At 7 d after HI injury, the HI group exhibited significantly lower synaptophysin levels than the sham group (*P* < 0.05), and the difference became more obvious at 14 d after HI injury (*P* < 0.01). From 14 d to 21 d after HI injury, there was a marked increase in synaptophysin, resulting in no significant difference between the two groups at 21 d after HI injury. However, the level of PSD-95 significantly decreased at 14 and 21 d after HI injury in the HI group, and no difference in PSD-95 expression between the two groups was identified at 3 d and 7 d after HI injury. The difference in time-dependent protein expression indicated that PSD-95 synthesis was later than that of synaptophysin during synaptogenesis.

**Figure 5:**
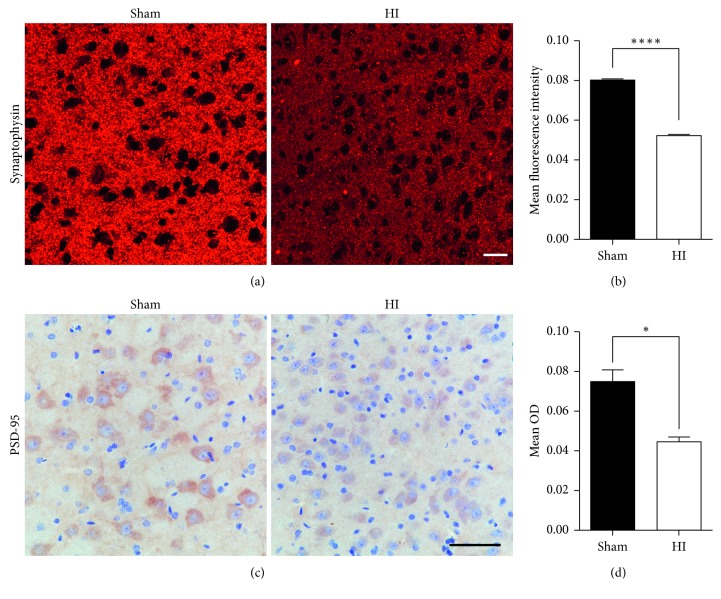
The changes of synaptophysin and PSD-95 expression in the thalamus exhibited by immunofluorescence and immunohistochemistry staining. (a, b) Immunofluorescence staining showed synaptophysin in the right thalamus at 14 d after HI injury (scale bar = 20 *μ*m). The quantitative analysis showed lower expression of the synaptophysin in the thalamus in HI rats than in that of sham rats. (c, d) Immunohistochemistry staining showed PSD-95 in the right thalamus at 14 d after HI injury (scale bar = 50 *μ*m). The quantitative analysis showed lower expression of the PSD-95 in the thalamus in HI rats (^*∗*^*P* < 0.05 and ^*∗∗∗∗*^*P* < 0.0001).

We also conducted immunofluorescent examination on synaptophysin and immunohistochemical examination on PSD-95 at 14 d after HI injury. It revealed a significant decrease in fluorescence intensity of synaptophysin in the thalamus of HI rats compared to sham rats (*P* < 0.0001, Figures 5(a) and 5(b)). The analysis of ODs indicated the lower expression level of PSD-95 in the thalamus of HI rats compared to sham rats (*P* < 0.05, Figures 5(c) and 5(d)).

## 4. Discussion

The degeneration and impaired maturation of OLs in the developing brain have been considered to be the main mechanisms of myelination failure associated with WMI induced by HI injury [1, 6–8, 10]. The present study showed that HI exposure in rats caused hypomyelination and OLs reduction, in agreement with results from other WMI studies [23, 24]. The brain region analyzed for the expression of MBP was CC, which was rich in myelin in the periventricular region. In perinatal WMI, decreased expression of MBP was considered to be associated with long-term cognitive dysfunction [25]. The lower MBP expression level was associated with axonal myelination insufficiency determined by electron micrography [26]. Hypomyelination in the CC and cortex will result in insufficient information processing and conduction velocity between cortical regions or between cortex and deep gray nuclei, such as the connections between the thalamus and cortex, which will be discussed in the following part. We further noted loss of synapses in the thalamus. In addition, ultrastructural synaptic features, such as AZ length and PSD thickness, were reduced at 2 weeks after HI injury.

Synapses play a vital role in information transduction between neurons and are essential for complicated signal processing and network organization in the nervous system [15, 16]. Impaired synapse physiology is involved in the pathogenesis of many neurodevelopmental and neurodegenerative diseases, such as autism [27], Down syndrome [28], epilepsy [29], Alzheimer disease [30, 31], and Parkinson disease [32]. In the present study, we noted a reduction in the numerical density and an increase in ultrastructural defects of synapses in the thalamus of rats with WMI induced by HI injury, probably leading to physiological and behavioral dysfunction [16, 27–33]. In addition, the thalamus has extensive connections with cortical regions and serves as a relay station for sensory information transduction to the cortex, participating in vision formation [11, 34]. The network connecting the thalamus and cortex displays regulatory properties in learning, memory, decision-making, and other cognitive behaviors [11, 35, 36]. The importance of connections was highlighted for its association with neurological disabilities [37]. The injury to the thalamus may involve multiple developmental domains, which is consisted with the neurological disabilities including adverse neurocognitive outcomes, visual dysfunction, disturbances in learning, memory, attention, and executive function in WMI survivors that cannot simply be induced by myelination failure. Hypomyelination accompanied by synaptic defects in the thalamus may provide a more comprehensive and reasonable pathological basis for the broad spectrum of disabilities observed in these patients. Thus, we choose the thalamus as a typical area for research, focusing on the synaptic defects in the thalamus. However, neuronal degeneration in the hippocampus and cerebellum was reported in some neuroimaging and neuropathological studies [12, 37]. The relation of the hippocampus and cerebellum deficits after HI injury with cognitive disability in WMI survivors needs further exploration.

Presynaptic membranes are primarily located on axon terminals, while postsynaptic membranes are located on dendritic spines. McClendon et al. found neuronal dysmaturation in response to HI injury in the caudate nucleus of fetal sheep, which manifested as a decrease in dendritic spines without neuronal death [38]. By performing quantitative volumetric and DTI measurements, Nagasunder et al. reported a volumetric reduction in the thalamus and thalamic microstructural abnormalities in PVL survivors, suggesting axon degeneration that likely contributed to cognitive visual processing disabilities [14]. The above two findings provide possible anatomic evidence for abnormal synaptic abnormalities in patients with WMI.

Extracellular matrix (ECM) molecules from reactive astrocytes are viewed as chemical barriers for synaptogenesis and synaptic plasticity [17, 39]. ECM molecules play vital roles in the early development of the nervous system by guiding neurite outgrowth, synaptogenesis, and synaptic plasticity. In WMI, accumulated reactive astrocytes surrounding the damaged area form glial scars and secrete neurotoxins and ECM molecules, among which hyaluronic acid (HA) and some chondroitin sulfate proteoglycans (CSPGs) have been found to be responsible for limiting neurite growth and axonal regeneration after injury, thereby impairing synaptogenesis and synaptic plasticity [39, 40]. Increased activity of the ECM molecule MMP9 has been associated with structural alterations of spines or altered synaptic plasticity [41]. In addition to ECM molecules, glycogen synthase kinase 3*β* (GSK-3*β*) has been shown to be involved in HI injury-induced synaptic abnormalities. Increased GSK-3*β* levels after ischemia and reperfusion exposure in rat brains were related to negative regulation of antioxidant ability and neuron death, suggesting possible relationships with synaptic abnormalities [42]. In addition, increased activity of GSK-3*β* likely promotes tau phosphorylation and neurodegeneration and, subsequently, synapse dysfunction [43, 44].

Ultrastructural changes in synapses, manifesting as decreased AZ length and PSD thickness at 2 weeks after HI injury exposure, were shown in the present study. The AZ, which is located on the presynaptic terminal, consists of scaffold proteins ensuring fast but adjustable neurotransmitter release, thus playing a prominent role in regulating the efficacy of neurotransmitter release [17, 45, 46]. In contrast, the PSD is formed by the scaffold of densely organized proteins that anchor neurotransmitter receptors and cell-adhesion molecules responsible for information transduction [47, 48]. The decreased AZ length and PSD thickness in the thalamus in the present study can be explained by HI injury and suggest impaired interneuronal connectivity and information transduction, which may subsequently result in motor defects or cognitive disability.

We found increased expression of synaptophysin and PSD-95 from 3 d to 21 d after HI injury in this study, suggesting continuous synaptogenesis during this period. The two groups demonstrated no significant difference in synaptic proteins expression at 3 d after injury. One of the possibilities is that damage to axon, which contains the presynaptic protein, may be induced by degeneration secondary to the death of neuronal cell bodies of thalamus [49]. The secondary injury may not be evident during the early days after HI injury. The delayed injury is important to preterm brain injury, as it results in impairments in the neonate that evolve into complicated disabilities over time [37]. Besides, dendritic spines, which contain the postsynaptic protein, are less in the immature neurons during the early 10 days after birth [50], consisted with the low expression of PSD-95 in sham rats from 3 d to 7 d after modelling in the present study. The injury to the spine is probably not significantly manifested at 3 d after injury. However, the HI group expressed significantly less of these two synaptic proteins at 14 d after injury than the sham group. The loss of synapses and decrease in AZ length and PSD thickness induced by HI injury provides some possible evidence for the above findings. PSD-95 is a member of the scaffold proteins in the PSD and is involved in the organization of postsynaptic signals and regulation of synaptic transmission [47, 48, 51]. The first identified synaptic vesicle (SV) membrane protein, synaptophysin, is required in the biogenesis of SVs and in the regulation of SV trafficking, which may be related to synaptic plasticity [45]. The decreased levels of synaptophysin and PSD-95 suggest impaired synaptic plasticity and signal transmission. The decreased expression of PSD-95 continued until 21 d after HI injury, at which point the levels of synaptophysin were not significantly different between the HI and sham groups. We proposed that the marked increase in synaptophysin from 14 d to 21 d after HI injury in HI rats may be a compensatory change that helps to improve the efficiency of transmitter release by promoting the biogenesis of SVs. However, this synaptic reorganization with an imbalance in presynaptic and postsynaptic protein expression, which has also been observed in immature brains with seizure-related injury [52], probably leads to uncoordinated synaptic transmission.

Hypomyelination in the thalamus was reported in some neuroimaging studies [14, 37]. We have not focused on the change of myelin in the thalamus in the present study. It could be a direction for our future research.

In the present study, we used a rat model of WMI and demonstrated that neonatal HI injury not only induced OL reduction and hypomyelination but also led to synaptic loss and ultrastructural and synaptic protein expression abnormalities. Importantly, we found synaptic reorganization induced by HI injury, which may aggravate synaptic dysfunction by disturbing signal transmission. Further investigations are needed to explore the mechanism of synaptic reorganization induced by HI injury and synaptic changes after HI injury over a longer period.

## Data Availability

The data used to support the findings of this study are available from the corresponding author upon request.
